# Temperature elevation in tissue detected *in vivo* based on statistical analysis of ultrasonic scattered echoes

**DOI:** 10.1038/s41598-020-65562-4

**Published:** 2020-06-03

**Authors:** Michio Takeuchi, Toshihiko Sakai, Gabor Andocs, Keizo Takao, Ryo Nagaoka, Hideyuki Hasegawa

**Affiliations:** 1 Medical Device Division, Tateyama Kagaku Industry Co., Ltd., 30 Shimonoban, Toyama Toyama, 930-1305 Japan; 2 Life Science Research Center, University of Toyama, 2630 Sugitani, Toyama Toyama, 930-0194 Japan; 3 Medical Business and Research Department, Tateyama Machine Co., Ltd., 30 Shimonoban, Toyama Toyama, 930-1305 Japan; 4 Department of Radiology, Faculty of Medicine, University of Toyama, 2630 Sugitani, Toyama Toyama, 930-0194 Japan; 5 Department of Behavioral Physiology, Faculty of Medicine, University of Toyama, 2630 Sugitani, Toyama Toyama, 930-0194 Japan; 6 Faculty of Engineering, Academic Assembly, University of Toyama, 3190 Gofuku, Toyama Toyama, 930-8555 Japan

**Keywords:** Medical research, Engineering, Physics

## Abstract

It is demanded to monitor temperature in tissue during oncological hyperthermia therapy. In the present study, we non-invasively measured the temperature elevation inside the abdominal cavity and tumour tissue of a living rat induced by capacitive-coupled radiofrequency heating. In the analysis of ultrasound scattered echoes, the Nakagami shape parameter *m* in each region of interest was estimated at each temperature. The Nakagami shape parameter *m* has temperature dependence; hence, the temperature increase inside tissue specimens can be detected with the *m* values. By carrying out *in vivo* experiments, we visualized the temperature increase inside the abdominal cavity and tumour tissue of living rats using two-dimensional hot-scale images indicating the absolute values of the ratio changes of the *m* values. In both the abdominal cavity and tumour tissue, the brightness in the hot-scale images clearly increased with increasing temperature. The increases in brightness in the hot-scale images imply the temperature elevations inside the abdominal cavity and tumour tissue of the living rats. The study results prove that the acoustic method we proposed is a promising method for monitoring changes in the internal temperature of the human body under hyperthermia treatment.

## Introduction

Non-invasive measurement of internal body temperature distribution has great potential in the medical field. In particular, internal body temperature distribution is the most important parameter for carrying out oncological hyperthermia therapy safely, correctly, and effectively. However, currently, hyperthermia therapy is conducted without monitoring internal body temperature, including the temperature of malignant tumour tissue, in almost all cases. There is no realistic method to non-invasively detect internal body temperature distribution during heating under therapy except for operating a magnetic resonance imaging (MRI) device. Although it is well known that internal body temperature can be detected by an MRI instrument^1–5^, an MRI device is adopted as an internal body temperature detector by only one hyperthermia device (BSD-2000 3D/MR; Pyrexar Medical, Salt Lake City, UT, USA)^6^. Unfortunately, this hyperthermia device is not preferred among oncological hyperthermia societies because of its high price. A major trend is that oncological hyperthermia therapy devices require an invasive temperature measurement system using a thermocouple or a fibre optic temperature sensor. However, nobody actually monitors the internal body temperature by inserting a sensor probe into the tumour tissue. A realistic method to non-invasively measure the temperature distribution inside the human body has been demanded by oncological hyperthermia societies for three decades.

In addition, many researchers have proposed a solution using ultrasound technologies instead of using an MRI instrument. There is a major engineering trend to detect internal body temperature based on the temperature dependence of ultrasound propagation speed^7–12^. Those studies have achieved success in detecting a temperature increase inside biological tissue in *in vitro* and *ex vivo* situations. However, the temperature increase can not be measured precisely by the acoustic method with ultrasound propagation speed in *in vivo* situations because body motion and pulsation exist. In this method, the temperature change is estimated with a known ultrasound propagation path length and propagation time. It is unrealistic to estimate precise axial displacement along the ultrasound propagation path using a B-mode image in an *in vivo* situation.

On the other hand, it was reported that some statistical properties obtained by statistical analysis of ultrasound backscattered echoes can be applied to quantify liver diseases^13–16^, detect scatterer size and density in a tumour^17–19^, and classify breast lesions^20,21^; hence, some research groups found that the statistical parameters obtained from analysis of ultrasound scattered echoes have the potential to detect temperature distribution inside biological tissue specimens^22–26^. Moreover, a computer simulation study has demonstrated that one of the statistical parameters, the Nakagami shape parameter *m*, is strongly related to the scatterer concentration in the medium^27^; thus, it is predicted that the Nakagami shape parameter *m* changes with a change in the medium volume due to thermal expansion or contraction of medium containing scatterers. Our research group has focused on the possibility of non-invasively monitoring the temperature distribution inside the human body with the temperature dependence of the Nakagami shape parameter *m* for oncological hyperthermia treatment. We previously reported phantom and *ex vivo* study results^22,23^. We presented that it is important to avoid the influence of deformation resulting from a temperature elevation in soft tissue specimens to select a proper size of a region of interest (ROI) in the estimation of the shape parameter of the Nakagami distribution^22^. In that study, temperature changes inside soft tissue with deformation could be detected as a two-dimensional hot-scale image indicating absolute values of ratio changes of *m* values, *α*, estimated with some ROI sizes that were assumed to be larger than the amount of displacement of the soft tissue^22^. The study result implies that internal temperature changes under *in vivo* conditions can be expressed as hot-scale images indicating absolute values of ratio changes of *m* values estimated by selecting proper ROI sizes with consideration of the displacement due to body motion and pulsation. Furthermore, it was shown in our previous study that variations in the Nakagami shape parameter *m* due to a change in temperature depend on an initial *m* value^23^. By taking into account the initial *m* value dependence, we proposed a new parameter *α*_mod._ that was elicited by adding the multiplying factor varying as a function of the initial *m* value to absolute values of ratio changes of *m* values, *α*^23^. Furthermore, the temperature gradient inside a locally heated real soft tissue specimen with no thermal lesions was visualized with two-dimensional *α*_mod._ maps more clearly than that visualized using the previous method with *α*^23^. In this study, we present an *in vivo* study result showing that the temperature elevation inside the abdominal cavity and tumour tissue of a living rat induced with capacitive-coupled radiofrequency (RF) current heating was detected by hot-scale images indicating absolute values of ratio changes of *m* values, *α*_mod._.

## Results

### Temperature elevation inside tissue detected in vivo by ultrasound scattered echoes in healthy rat

**Figure 1:**
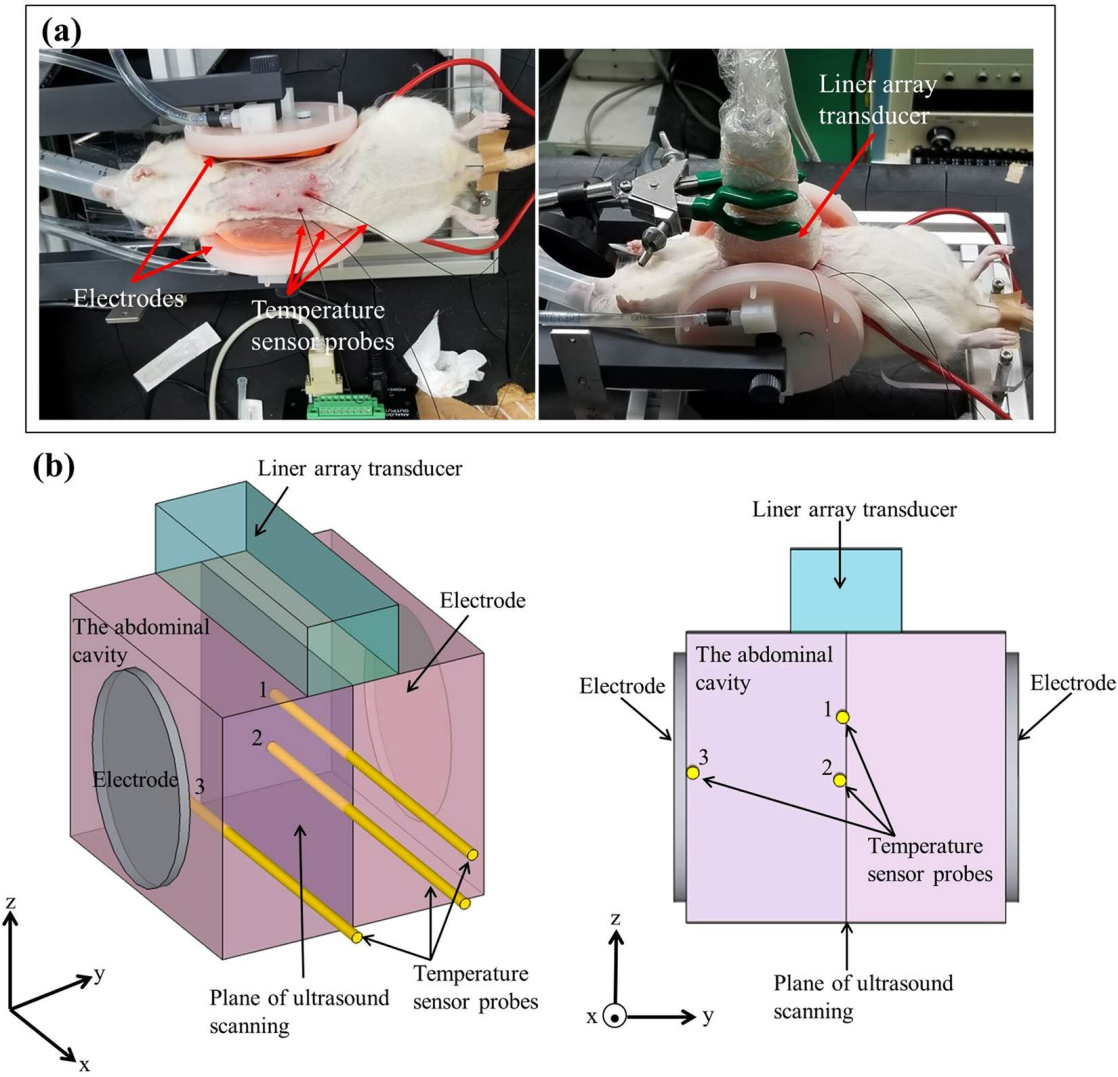
(**a**) Photograph of the experimental setup for the healthy rat experiment. **(b)** Schematic of the experimental setup for the healthy rat experiment.

**Figure 2:**
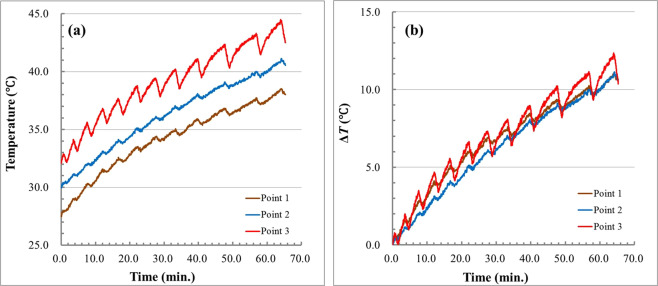
(**a**) Variations in the temperature inside the abdominal cavity and on the surface of the skin of the rat. **(b)** Temperature increase inside the abdominal cavity and on the surface of the skin from non-induced state Δ*T* as a function of the reference temperature.

In the healthy rat experiment, an Slc:SD female living rat was heated from 30.0 to 41.0 °C by RF current. Ultrasound scattered echoes from the abdominal cavity of the rat were measured. In addition, temperatures inside the abdominal cavity were detected with fibre optic temperature sensor probes. Figure 1(a,b) show the experimental setup and the schematic of the experimental setup for the healthy rat experiment. In the healthy rat experiment, the temperature measured at Point 2 is defined as a reference temperature. The temporal variations in temperature inside the abdominal cavity and on the surface of the skin under the electrode of the living rat during heating are shown in Fig. 2(a). As seen in Fig. 2(a), temperatures at each point varied with ripples due to the RF current generator being paused briefly each time ultrasound scattered echoes were measured. The temperature increase from the non-induced state Δ*T* at each point is plotted as a function of the reference temperature in Fig. 2(b).

**Figure 3:**
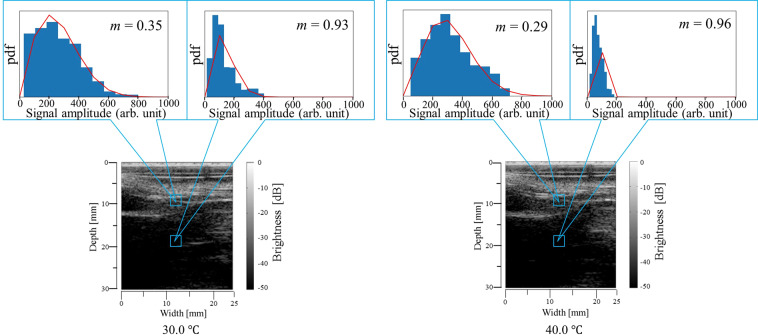
Grey-scale B-mode images of the abdominal cavity at reference temperatures of *T* = 30.0 and 40.0 °C and representative histograms of envelopes obtained by setting the ROI size at 1.8 × 1.8 mm^2^ and fitting the Nakagami distribution function to the histogram. The red solid line indicates the Nakagami distribution function.

Figure 3 shows typical grey-scale B-mode images obtained from the abdominal cavity of the rat at reference temperatures of 30.0 and 40.0 °C and histograms of envelopes of the ultrasonic echo signal. The red solid line indicates the Nakagami distribution function in Fig. 3. In the B-mode images, there is a non-negligible deformation between each reference temperature. The displacement seen in the B-mode images is thought to be due to body motion or pulsation during heating. In our previous study^22^, it was presented that the calculation of absolute values of ratio changes of *m* values can be done correctly by selecting a proper ROI size even when deformations are observed in biological tissue specimens. Thus, we carried out statistical analysis estimating the Nakagami shape parameter *m* and absolute values of ratio changes of *m* values, *α*_mod._, with a relatively large ROI set at 1.8 × 1.8 mm^2^ to prevent the effect of displacement caused by pulsation and body motion in this study, thereby creating156 ROIs for statistical analysis. The Nakagami shape parameter *m* was estimated by statistical analysis as follows. First, analytic signals were elicited by applying the Hilbert transformation to measured ultrasonic RF signals, and envelope signals were obtained. Then, the histograms of envelopes in each ROI were created by setting the ROI size at 1.8 × 1.8 mm^2^. The Nakagami distribution function is expressed by

1 $${{f}}_{{N}}({r})=\frac{2{{m}}^{{m}}{{r}}^{2{m}-1}}{\Gamma ({m}){\Omega }^{{m}}}\exp \left(-\frac{{\rm{m}}}{\Omega }{{r}}^{2}\right){\boldsymbol{U}}({r}),$$

where Γ(∙) and *U*(∙) are the gamma function and unit step function, respectively, *r* is the amplitude of envelopes of ultrasound scattered echoes, *m* is the Nakagami shape parameter, and Ω is a scaling parameter. Finally, the Nakagami shape parameter *m* for each ROI was estimated by fitting the Nakagami distribution function to the histogram of the envelope of the ultrasonic scattered echoes (see Fig. 3). The normalized mean squared error (NMSE) was calculated to evaluate the goodness of fitting of the Nakagami distribution function to the histograms. NMSE was calculated as

2 $${\rm{N}}{\rm{M}}{\rm{S}}{\rm{E}}=\frac{\frac{1}{M}{\sum }_{{\boldsymbol{i}}=1}^{M}\,{\{h({r}_{i})-{f}_{N}({r}_{i})\}}^{2}}{\frac{1}{M}{\sum }_{{\boldsymbol{i}}=1}^{M}\,{f}_{N}{({r}_{i})}^{2}},$$

where *M* and *h*(*r*_*i*_) are the number of bins and the height of bin at each amplitude of envelopes of ultrasound scattered echoes. Mean values of NMSE of 156 ROIs at each reference temperature are listed in Table 1. The mean value of NMSE for the abdominal cavity is approximately 0.15. Meanwhile, the mean value of NMSE for the tumour tissue is approximately 0.04. The mean values of NMSE for the abdominal cavity and the tumour tissue are suitably small. In the study of Gambin and Kruglenko^24^, the goodness of fitting of the Nakagami distribution function to histograms of envelopes of ultrasonic RF signals scattered from biological tissue specimens was evaluated with NMSE. In the cited study, the values of NMSEs were from approximately 0.12 to 0.22. At the same time, in the present study, internal temperature changes in the biological tissue specimens were well detected using the Nakagami shape parameter. Therefore, the Nakagami distribution was considered to be a suitable approximation model.

To express the temperature increase inside the abdominal cavity by the variation of brightness on a two-dimensional hot-scale image indicating absolute values of ratio changes of *m* values, the specific parameter *α*_mod._ was calculated using

3 $${{\alpha }}_{mod.}=|{\gamma }\cdot {\log }_{10}\left(\frac{{{m}}_{{T}}}{{{m}}_{{{T}}_{{R}}}}\right)|,$$

where *m*_*T*R_ and *m*_*T*_ are the Nakagami shape parameters *m* at a baseline temperature and each temperature, respectively, and *γ* denotes the multiplying factor. In our previous *ex vivo* study^23^, it was clearly shown that the magnitude of the change in the Nakagami shape parameter (Δ*m*) due to a temperature rise has a dependence on the initial *m* value at a baseline temperature; furthermore, Δ*m* due to a temperature rise increases with increasing initial *m* value. Therefore, the ratio changes of *m* values were amplified with compensation considering the initial *m* values. In this study, the multiplying factor *γ* varying as a function of the initial *m* value was defined to be proportional to *m*^−1^ as

4 $${\gamma }({{m}}_{{{T}}_{{R}}})=\frac{10}{{{m}}_{{{\boldsymbol{T}}}_{{\bf{R}}}}}$$

**Figure 4:**
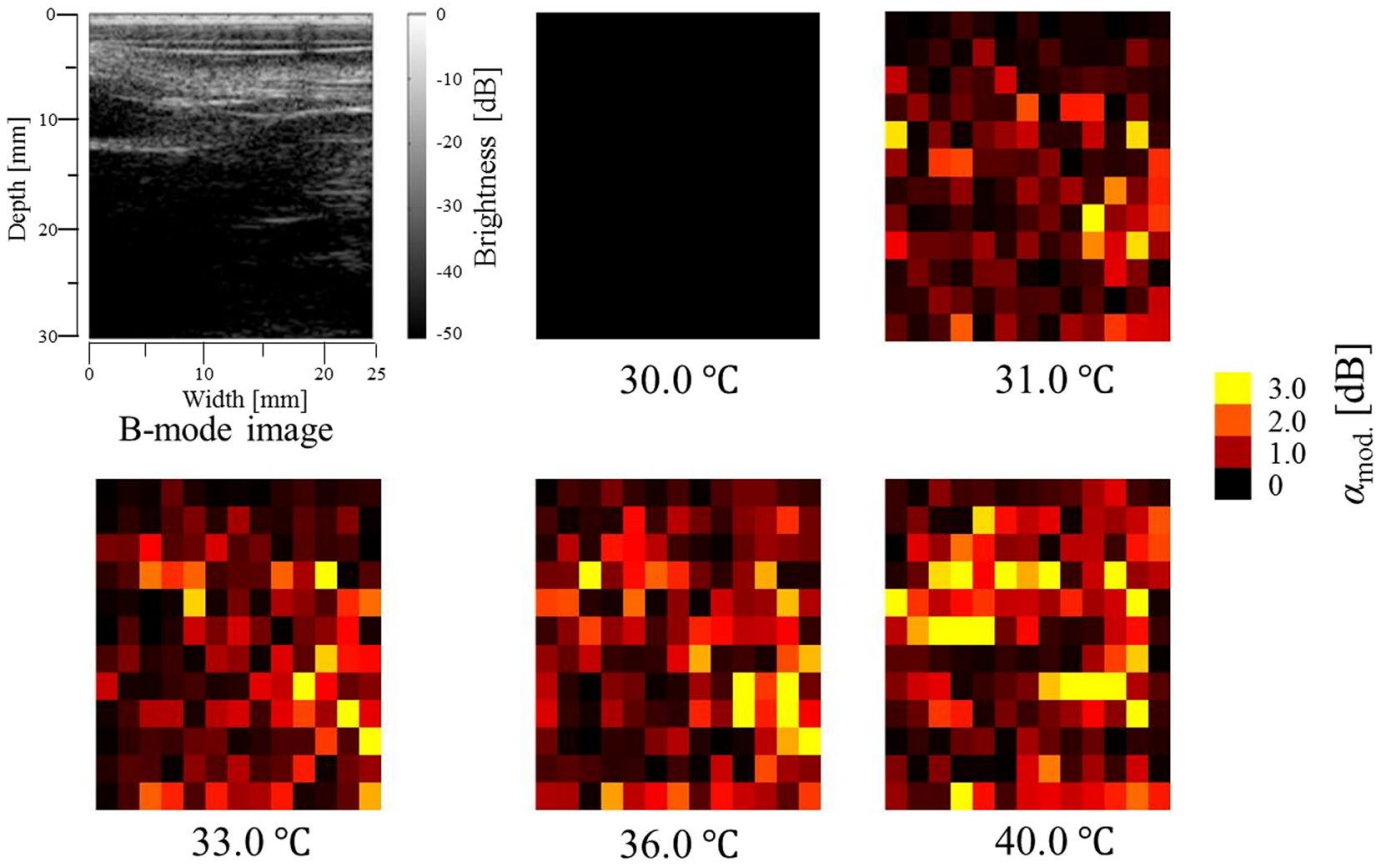
Grey-scale B-mode image of the abdominal cavity and hot-scale images indicating absolute values of ratio changes of *m* values, *α*_mod._, estimated by setting the ROI size at 1.8 × 1.8 mm^2^ for the abdominal cavity at each reference temperature.

The hot-scale images indicating the absolute values of ratio changes of *m* values, *α*_mod._, for the abdominal cavity calculated using Eqs. (3) and (4) with the baseline temperature of *T*_R_ = 30.0 °C are shown in Fig. 4. Note that the hot-scale images indicating the absolute values of ratio changes of *m* values, *α*_mod._, were constructed by comparing the Nakagami shape parameter *m* at a baseline temperature to *m* at each temperature estimated in each ROI with an overlap ratio of 0% between ROIs. In the hot-scale images, the overall increase in *α*_mod._ brightness with increasing reference temperature is clearly observed. The increase in *α*_mod._ brightness implies a temperature elevation in the abdominal cavity induced by RF current.

### Temperature elevation inside tumour tissue detected in vivo by ultrasound scattered echoes

**Figure 5:**
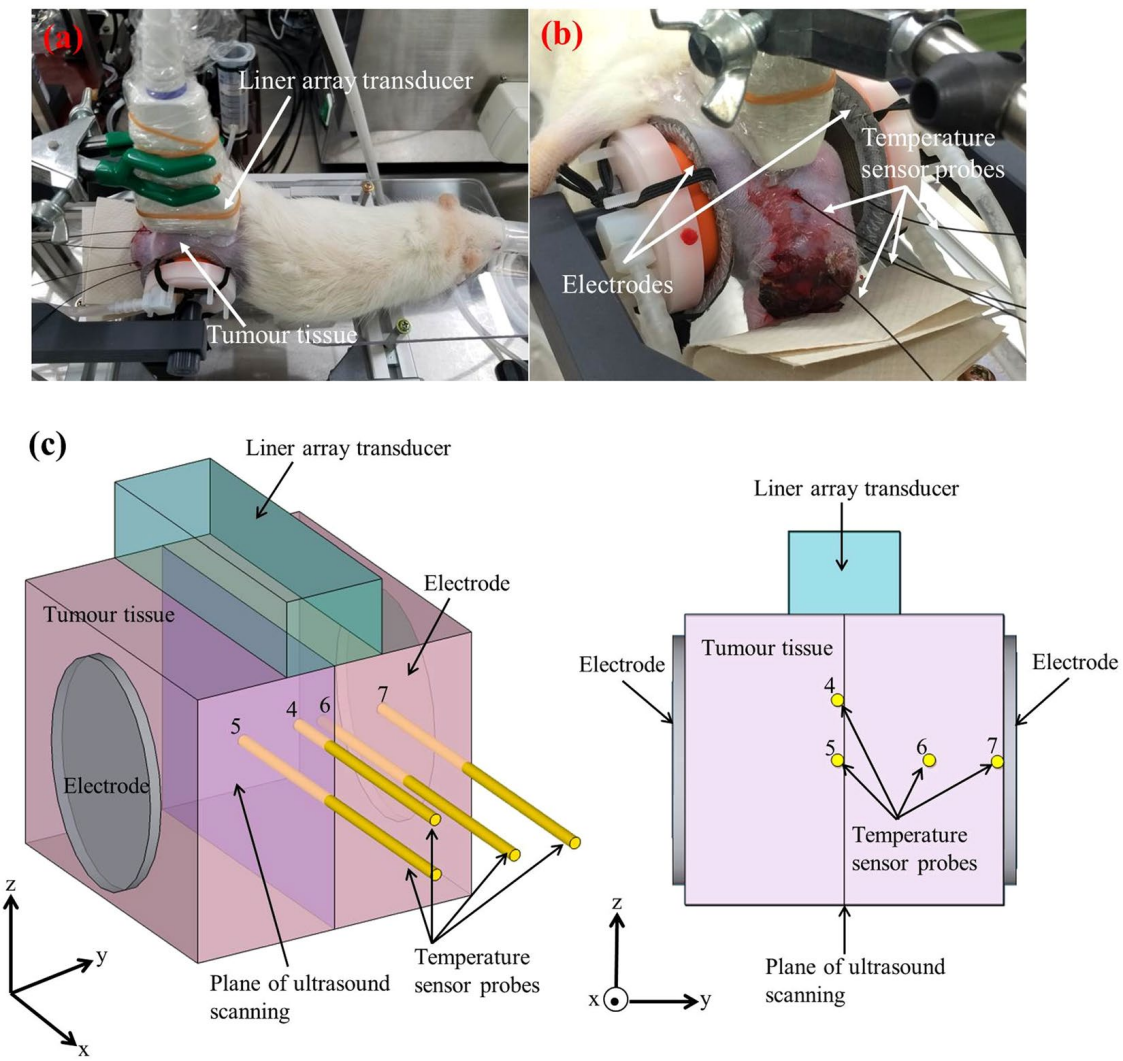
(**a**) Photograph of the experimental setup for the tumour tissue experiment. **(b)** Close-up photograph of tumour tissue. **(c)** Schematic of the experimental setup for the tumour tissue experiment.

**Figure 6:**
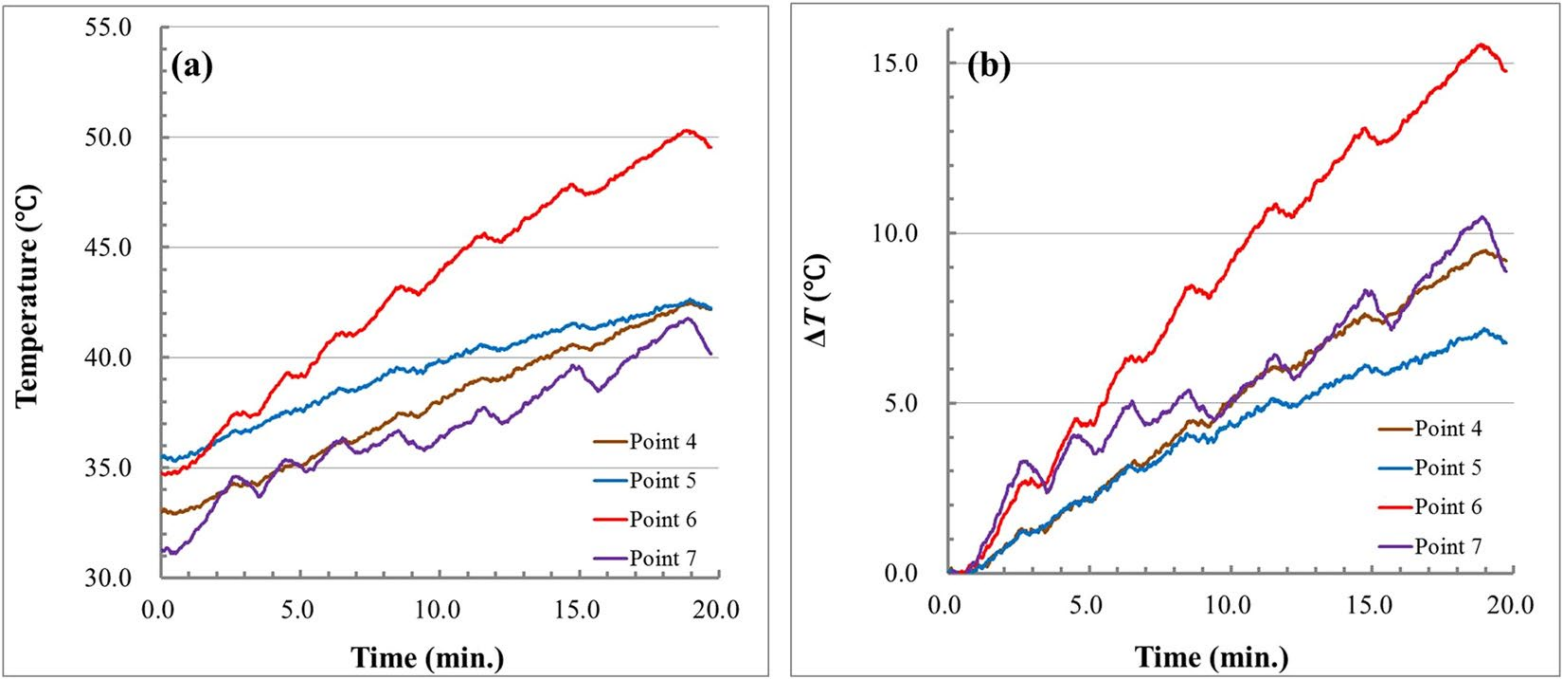
(**a**) Variations in the temperature inside the tumour tissue and on the surface of the skin of the rat. **(b)** Temperature increase inside the tumour tissue and on the surface of the skin from non-induced state Δ*T* as a function of the reference temperature.

In real therapy treatments, the oncological hyperthermia device is applied to the tumour tissue. Therefore, an experiment to detect temperature increases inside tumour tissue should be conducted in our study. In the experiment on tumour tissue, heterotopic tumour tissue was grown around the right femoral region of a Slc:SD female rat. Ultrasound scattered echoes from the tumour tissue were measured while the tumour tissue was heated from 35.5 to 42.5 °C. The experimental setup, a close-up photo around the tumour tissue, and the schematic of the experimental setup for the tumour tissue experiment are shown in Fig. 5(a–c). In the tumour tissue experiment, we used temperatures measured at Point 5 as a reference temperature. Figure 6(a) shows the variations in the temperature inside the tumour tissue and the surface of the skin of the rat during heating. The temperature increase from the non-induced state Δ*T* at each point is plotted as a function of the reference temperature in Fig. 6(b). In Fig. 6(b), the Δ*T* distribution inside the tumour tissue is intricate, not systematic, as expected from the positional relationship between electrodes and the tumour tissue. Δ*T* at point 6 increases steeply in Fig. 6(b). Moreover, Δ*T* at point 6 is approximately one and half times larger than Δ*T* at points 4 and 5. A computer simulation study predicted that the temperature distribution inside muscle, tumour tissue, brain, and some organs induced non-locally by electrical energy under *in vivo* conditions is significantly complex, and hotspots occur^28^. It is presumed that non-uniform heating is caused by inhomogeneous dielectric and/or thermal properties of organs and muscle. Hence, it is thought that the tumour tissue was heated up non-uniformly by the RF current and that a few hotspots existed inside the tumour tissue in this study.

**Figure 7:**
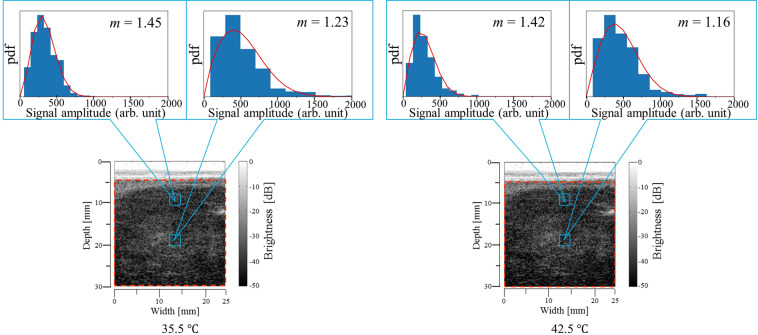
Grey-scale B-mode images of tumour tissue at reference temperatures of *T* = 35.5 and 42.5 °C and representative histograms of envelopes obtained by setting the ROI size at 1.8 × 1.8 mm^2^ and fitting the Nakagami distribution function to the histogram. The red solid line indicates the Nakagami distribution function.

**Figure 8:**
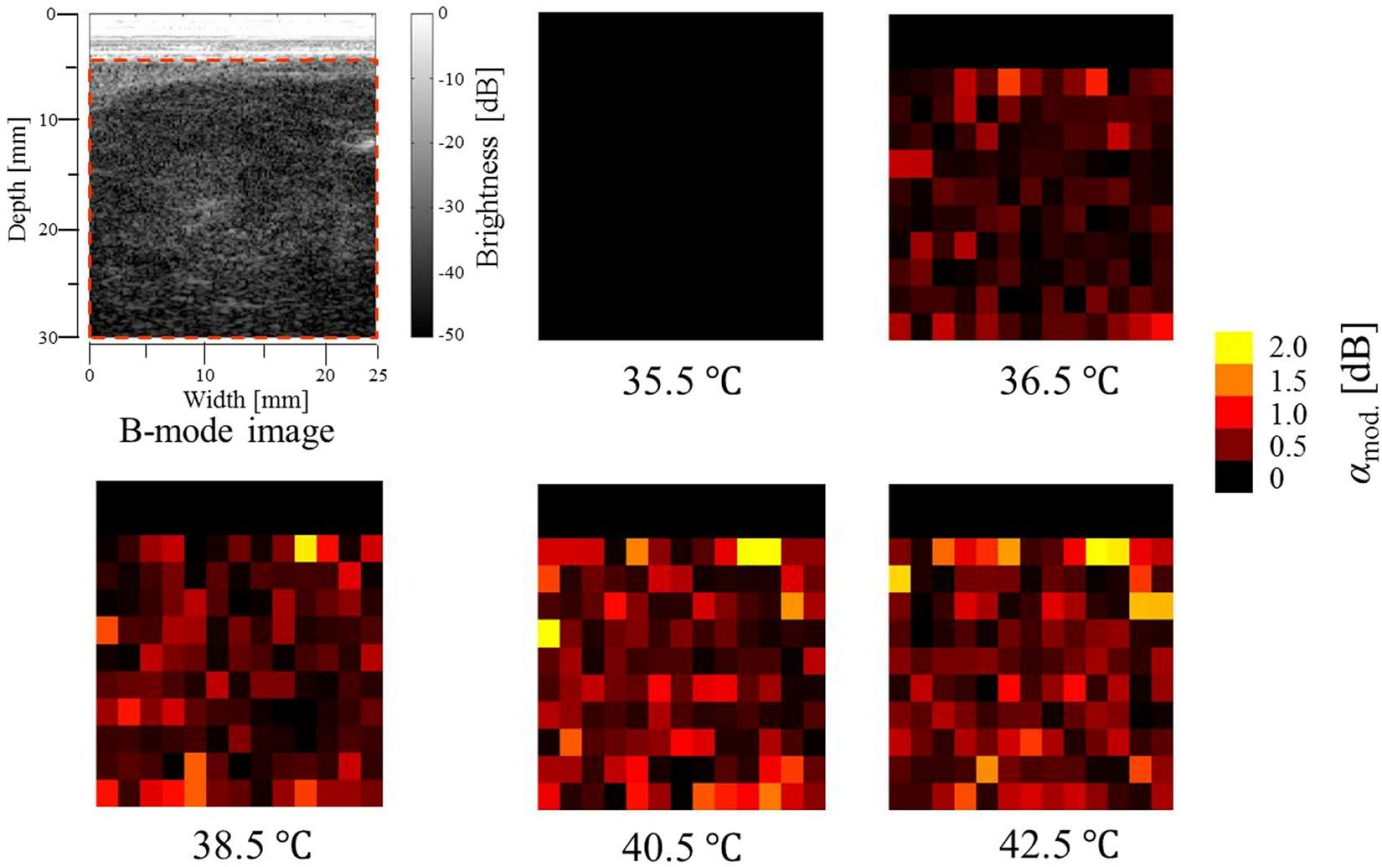
Grey-scale B-mode image of tumour tissue and hot-scale images indicating absolute values of ratio changes of *m* values, *α*_mod._, estimated by setting the ROI size at 1.8 × 1.8 mm^2^ for the tumour tissue at each reference temperature.

Grey-scale B-mode images obtained from the tumour tissue at reference temperatures of 35.5 and 42.5 °C and histograms of envelopes with the Nakagami distribution function fit (red solid line) at different ROIs are shown in Fig. 7. The shallow region below approximately 5 mm in the B-mode images for the tumour tissue experiment is indistinct. In the tumour tissue experiment, we conducted a statistical analysis of ultrasound scattered echoes on the area enclosed with the dotted red line in Fig. 7. The hot-scale *m*-parameter images for the tumour tissue drawn by setting the baseline temperature at 35.5 °C are shown in Fig. 8. In the hot-scale images, the overall temperature elevation inside the tumour tissue with increasing reference temperature was observed as an increase in the *α*_mod._ brightness as well as the results of the healthy rat experiment.

## Discussion

**Figure 9:**
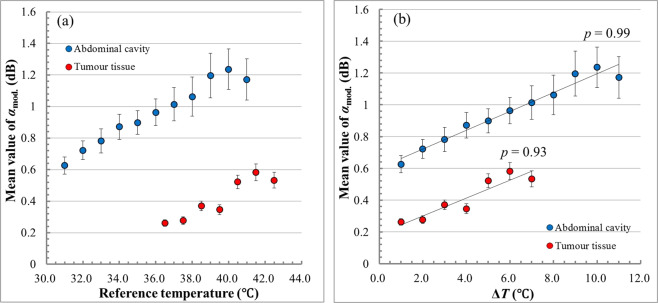
(**a**) Mean value of *α*_mod._ plotted as a function of reference temperature for the abdominal cavity and tumour tissue. **(b)** Δ*T* dependence of the mean value of *α*_mod._ for the abdominal cavity and tumour tissue.

The hot-scale images indicating the absolute values of ratio changes of *m* values, *α*_mod._, show the overall increases in temperature inside the abdominal cavity and the tumour tissue of the living rats. To evaluate the increase in *α*_mod._ with increasing temperature inside the abdominal cavity and the tumour tissue quantitatively, the mean value of *α*_mod._ on the analysis area of the ultrasonic RF signal was calculated at each reference temperature. Figure 9(a) shows the mean values of *α*_mod._ for the abdominal cavity of the healthy rat and the tumour tissue plotted as a function of the reference temperature. The error bar indicates the standard error of the mean of *α*_mod._ in Fig. 9(a,b). The mean value of *α*_mod._ for both the abdominal cavity and the tumour tissue monotonically rises with an increase in the reference temperature. Figure 9(b) shows the Δ*T* dependence of the mean value of *α*_mod._ for the abdominal cavity and the tumour tissue. We calculated the Pearson correlation coefficient *p* between the mean value of *α*_mod._ and Δ*T*. The coefficient of correlation *p* shows impressively large positive values, which are 0.99 for the abdominal cavity and 0.93 for the tumour tissue. An increase in *α*_mod._ indicated a temperature elevation inside the abdominal cavity, and the tumour tissue heated by RF current was confirmed quantitatively by the strong correlation between *α*_mod._ and Δ*T* in this study.

**Figure 10:**
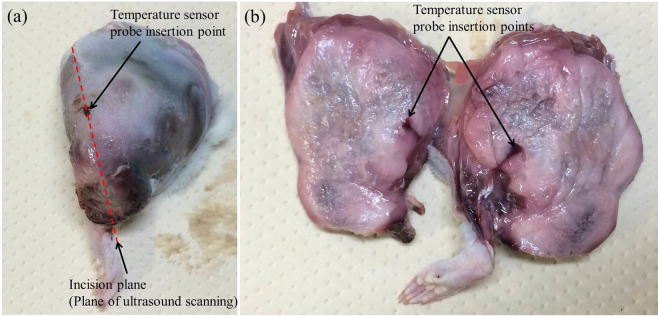
(**a**) Close-up photograph of the tumour tissue. **(b)** Cross-section of the tumour tissue.

Finally, since it was reported that a thermal lesion in soft tissue could be detected by changes in the Nakagami shape parameter^29^, it should be clarified that the increase in *α*_mod._ was not caused by heat-induced denaturation inside the tumour tissue due to the RF current being passed in this study. To observe the cross-section of the tumour tissue, the tissue was cut along the plane of ultrasound scanning after we completed the experiment. Figure 10(a) shows a close-up photo of the tumour tissue that was separated from the rat body. The cross-section of the tumour tissue is shown in Fig. 10(b). No visible transmutation was observed on the cross-section of the tumour tissue. Therefore, it can be concluded that the temperature elevation inside the tumour tissue of a living rat induced by RF current heating was detected by the hot-scale images, indicating the absolute values of ratio changes of *m* values.

In this study, we propose that our acoustic method is useful for detecting temperature elevations in tumour tissue heated by RF current *in vivo*. In our future study, the quantitative relationship between *α*_mod._ and Δ*T* should be investigated using a wide variety of tumour tissues to establish a method to measure the absolute value of Δ*T in vivo*.

## Methods

In this study, we conducted *in vivo* experiments using two Slc:SD female rats, which weighed approximately 0.3 kg. One was arranged for an experiment measuring ultrasound scattered echoes from the abdominal cavity (healthy rat experiment), and the other was arranged for an experiment with malignant tumour tissue (tumour tissue experiment). In the tumour tissue experiment, we prepared 9 L (glioma) cell line-derived heterotopic tumour tissue grown around the right femoral region of the Slc:SD female rat. The rats were anaesthetized by an inhalation anaesthesia system using isoflurane during the heat treatment in both experiments. In the healthy rat experiment, the abdominal cavity was heated from 30.0 to 41.0 °C by the energy of a capacitive-coupled RF current at a frequency *f* = 13.56 MHz between two flexible round-shaped electrodes holding both sides of that rat’s body. In the tumour tissue experiment, the tumour tissue was heated from 35.5 to 42.5 °C by passing an RF current at the frequency *f* = 13.56 MHz between the two electrodes holding the rat’s right hind limb including the tumour tissue. The electrodes were connected to a custom-built RF generator unit through an impedance matching circuit. The electrodes were water cooled to prevent surface overheating. Reference temperatures inside the abdominal cavity and the tumour tissue were measured by inserting fibre optic temperature sensor probes (m3300; LumaSense Technologies, Santa Clara, CA, USA). Ultrasonic echoes scattered from the heated specimens were measured at 1.0 °C intervals using an ultrasonic measurement system (RSYS0002; Microsonic, Kokubunji, Tokyo, Japan) with a linear array transducer (UST-5412; Hitachi-Aloka Medical, Mitaka, Tokyo, Japan). The two dimensional size of the scanning area in the specimens was approximately 30 mm in depth and 25 mm in width. The parameters in the ultrasonic measurement are listed in Table 2. The RF generator emits substantial electromagnetic waves, and it is strong enough to affect the piezoelectric elements of the transducer. The RF generator was paused emitting electromagnetic waves during acquisitions of ultrasonic echoes at the respective temperatures. The experimental setups for the healthy rat experiment and for the tumour tissue experiment are shown in Figs. 1(a) and 5(a). A close-up photo around the tumour tissue is shown in Fig. 5(b). Figures 1(b) and 5(c) show the schematic of the experimental setups for the healthy rat experiment and tumour tissue experiment. Points 1–7 in Figs. 1(b) and 5(c) indicate the positions of the tip of each sensor probe in the experimental setups for the healthy rat and tumour tissue experiments. In this study, we used temperatures measured at Points 2 and 5 as a reference temperature for the healthy rat experiments and tumour tissue experiments. It should be noted that we measured ultrasound scattered echoes at 1.0 °C intervals by referring to the reference temperatures. The application of RF current was stopped manually every 1.0 °C during acquisition of ultrasonic echo signals. After *in vivo* experiments, hot-scale images of temperature elevation inside specimens were processed by conducting statistical analysis of ultrasonic scattered echoes with custom-made software based on MATLAB R2018b (The MathWorks, Natick, MA, USA, https://www.mathworks.com/) and Python 3.6.0 (Python Software Foundation, DE, USA, https://www.python.org/).

**Table 1:** Mean values of NMSE for the abdominal cavity and tumour tissue.

Abdominal cavity		Tumour tissue	
Reference temperature (°C)	NMSE	Reference temperature (°C)	NMSE
30.0	0.1495	35.5	0.0428
31.0	0.1519	36.5	0.0428
32.0	0.1550	37.5	0.0413
33.0	0.1613	38.5	0.0402
34.0	0.1577	39.5	0.0417
35.0	0.1582	40.5	0.0427
36.0	0.1523	41.5	0.0418
37.0	0.1466	42.5	0.0414
38.0	0.1615		
39.0	0.1398		
40.0	0.1468		
41.0	0.1314

**Table 2:** Parameters of ultrasonic measurement.

Pitch of scan lines	200 μm
Number of scan lines	121
Number of samples per scan line	1200
Central frequency	7.5 MHz
Wavelength	0.2 mm
Sampling rate	31.25 MHz

### Ethical approval

All experiments and procedures involving animals were conducted in accordance with the Institutional Animal Experiment Handling Rules as approved by the Institutional Animal Care and Use Committee at University of Toyama (A2017OPR-2).

## Data Availability

The datasets during and/or analysed during the current study are available from the corresponding author on reasonable request.
